# Trends in Dyslexia Research during the Period 1950 to 2020—Theories, Definitions, and Publications

**DOI:** 10.3390/brainsci12101323

**Published:** 2022-09-29

**Authors:** Turid Helland

**Affiliations:** Department of Biological and Medical Psychology, Faculty of Psychology, University of Bergen, P.O. Box 7807, NO-5020 Bergen, Norway; turid.helland@uib.no

**Keywords:** dyslexia research, 1950–2020, definitions, symptomatic level, cognitive level, biological level environmental level, sensory motor skills, comorbidities, publication counts

## Abstract

Introduction. The focus of the present paper is on (1) how dyslexia research and hence definitions have developed during the period 1950–2020 and includes (2) a database search of scientific publications on dyslexia during the same period. The focus is on the definitions of dyslexia and the organization of the network search based on the causal four-level model by Morton and Frith. Method. (1) The definitions are presented in accordance with a historic review of dyslexia research from 1950 to 2020 and based on (2) Google Scholar counts of publications on dyslexia, on defining dyslexia, on dyslexia at the four levels (symptomatic, cognitive, biological, environmental), and by areas (sensorimotor, comorbidity). Finally, a percentage calculation shows the relative development within each level and area by decennium (1950–1960, 1960–1970, 1970–1980, 1990–2000, 2002–2010, 2010–2020). Results. (1) Of the seven definitions presented, only the definition by the BDA 2007 included the four levels of the causal model. (2) The number of publications increased substantially over the period. However, relatively few publications have defined dyslexia. An increase in publications from 1950 to 2020 was seen across the four levels and two areas—however, with an alteration in the thematic focus over this time span. Summary. Defining dyslexia has still not reached a consensus. This uncertainty may explain why only one of the seven definitions proved satisfactory according to the four-level model. Along with the general increase in research, publications on dyslexia have increased accordingly during the period 1950 to 2020. Although the symptomatic level has played a dominant role over the whole period, thematic shifts have been seen over these 70 years. In particular, a substantial thematic shift was seen by the turn of the millennium. There has been a relative increase in the focus on literacy at the symptomatic level, on phonological awareness at the cognitive level, in gender at the biological level, and second language learning as comorbidities. However, increases in counts are not alone a valid indication of scientific progress. In particular, the lack of definitional criteria as a basis for participant and method selection should attract much more focus in future studies. The present study underlines the multifactorial nature of dyslexia, as evidenced by a substantial increase in the number of publications on the subject. It is a challenge for future research to continuously use and possibly redefine dyslexia definitions in line with such standards.

## 1. Part I—A Historical Overview

More than a hundred years ago, some curious cases of dyslexia were detected in gifted boys, and there were doctors who described these cases of “word blindness” in medical journals. Today, dyslexia is a term that is commonly known and denotes an innate disposition leading to reading and/or writing difficulties. Medicine, linguistics, psychology, pedagogics, social sciences have been involved in assessing dyslexia from different angles. This study aimed to follow this development by decades from 1950 to 2020. The introductory part is a brief historical overview of the theories and research during this period, while the second part is a network search on the counts of dyslexia scientific publications during this period of time.

### 1.1. The Pioneers

As the art of reading became widespread and common, the condition “word blindness” was also observed and described at the end of the 19th century by medical doctors. The condition was characterized as dramatic because the distance between the writing skills on the one hand and the intellectual abilities on the other was great. The doctors related these weak skills to language functions localized in the angular gyrus in the left hemisphere of the brain [1,2,3].

The American medical doctor Samuel Torrey Orton (1879–1948) called the difficulties “strephosymbolia”, which means “twisted symbols” [4]. He had observed that many of the children he worked with tended to reverse the letters or read and write them in the wrong order. In his research, he eventually focused on language difficulties, studying over three thousand children and adults with such difficulties. This led to a theory that the children’s reading difficulties could be explained by the fact that the left hemisphere of the brain did not become dominant in relation to the right hemisphere of the brain. He also pointed out that the difficulties were familial.

Based on this, Orton wanted to develop a way to learn to read and write as integrated right- and left-brain functions. He started a school based on this idea, which consisted of “multisensory” training. This is a combination of kinesthetic (motion-based) and tactile (touch or sensory) learning strategies integrated with the learning of visual and auditory concepts [5].

Right up until the 1960s, children who did not learn to read and write as expected were seen as single cases, either as developmentally disabled or as non-educationally skilled. Some were taken under the protective wings of concerned teachers who saw these children and gave them extra instruction, often after the end of the school day.

### 1.2. 1950–1970

#### 1.2.1. From Medicine to Educational Psychology Approach

Eventually, dyslexia was studied not only by doctors but also by psychologists, sociologists, and pedagogues. This led to a conflict of competence between researchers and clinicians who evoked new theories about the symptoms and causes of dyslexia. The backdrop to what occurred was the so-called “cognitive revolution”—a reaction to behaviorism, which emphasized that psychological science could only base itself on observable factors. Fundamental to the cognitive revolution was the idea that human activities are complex processes that require organization and planning. Noam Chomsky developed the theory of universal grammar in the 1950s [6]; Jean Piaget developed his well-known theories about the cognitive development of the child through stages [7]; Lev Vygotskij pointed out the importance of culture to the child’s development [8]; and George Miller launched his theories on information processing [9]. This led clinicians, sociologists, and educators to discuss how the environment through pedagogical methods could affect the dyslexic pupil’s skills and difficulties. In 1968, the World Federation of Neurology (WFN) defined dyslexia as follows:

*“A disorder manifested by difficulty in learning to read despite conventional instruction, adequate intelligence, and sociocultural opportunity. It is dependent upon fundamental cognitive disabilities which are frequently of constitutional origin.”*

[10,11]

This definition has been criticized as being “an exclusion definition”, which refers to the fact that, for example, underprivileged people or people with low IQ could be excluded from being able to receive a dyslexia diagnosis. Despite this criticism, the definition has been used as a criterion for subject selection, especially in medically oriented research. In a research context, this serves a purpose of avoiding cluttering comorbidities, but in clinical work, the consequences have been more debatable.

Wechsler’s intelligence tests, published in the period 1949 to 1967, are probably the tests that have been most commonly used to map cognitive profiles in both clinical and research settings. The typical dyslexic IQ profile has been presented as low scores in the following four subtests: Arithmetic, Coding, Information, and Digit pan, abbreviated to the so-called ACID profile. Thus, the researchers would naturally look for an ACID profile when investigating possible dyslexia, compatible with the WNF’s definition. In practice, this meant that people with an IQ score lower than 85 points, which includes 17% of the population, could not be diagnosed with dyslexia. For an overview of the tests, please see Frank [12].

#### 1.2.2. Groupings by Function Analysis

In the 1960s, Johnson and Myklebust focused on children who had problems with information processing, which could inhibit comprehension, speech, reading, mathematical or logical reasoning [13]. The difficulties observed in reading and writing were categorized. Visual dyslexia characterized the difficulties of those who were unable to learn words as a whole or had difficulties with visual discrimination. Difficulties in reversing words or letters while reading or writing were a typical symptom of this group. Auditory dyslexia characterized the difficulties of those who failed to synthesize the phonemes they heard into a visual component like a letter or a word.

Later, Boder extended this grouping to three different types of dyslexia. Dysphonetic dyslexia reflects deficits in associating symbols with sounds [14]. Typical reading errors would include letters being omitted, or the meaning of the word being distorted, and typical misspellings would be of a sound nature, e.g., the /t/ and /g/ being pre-exchanged. Dyseidetic dyslexia reflects a deficit in the ability to perceive letters and whole words as configurations, or visual gestalts, but not difficulties with the auditory processing. Mixed dysphonetic–dyseidetic dyslexia is a combination of both types of difficulties and difficulties in perceiving letters and words as visual gestalts.

### 1.3. 1970–2000

#### 1.3.1. Deficits within the Phonological System

A turning point in the view of subgroups of dyslexia came with research, which refuted that dyslexia could be explained by difficulties in remembering sequences of visual symbols—so-called visual dyslexia [15,16,17]. Instead, Vellutino pointed out that much research supports the theories that dyslexia (reading disability) is due to either difficulties with one or more aspects of linguistic functions or specific difficulties with visual/verbal integration. This represented the beginning of the explanation model, which has been dominating for a long time, namely that dyslexia is closely related to a difficulty in processing linguistic elements. By testing phonological awareness, rapid phonological retrieval, and phonetic transcoding in working memory, Snowling and Hulme found that people with dyslexia had special difficulties with what was collectively referred to as phonological processing [18]. Phonological awareness (PA) is defined as a conscious access to the sound components of the spoken language and the ability to manipulate these sounds. Phonological awareness is a metalinguistic skill that requires conscious attention and reflection on linguistic structures. Phonological awareness is therefore associated with other skills, such as general cognitive abilities, verbal short-term memory, and perception of speech [19].

The linguistic approach to dyslexia contributed to questioning the validity of the discrepancy criteria from the 1970s and 1980s. The Connecticut Longitudinal Study identified a representative sample of 445 preschool children and followed them for many years. An important finding was that children aged 6 to 18, who either had a discrepancy between IQ and reading scores or no such discrete but low reading scores, showed no difference in reading development compared to a control group [20].

#### 1.3.2. Dyslexia and Access to Lexicon: The Theory of the Double Deficit

A combination of difficulties within the phonological system, which is shown both by misproduction and difficulty with rapid automatized naming (RAN), has proven to be characteristic of people with major dyslexic difficulties, regardless of the linguistic affiliation. According to some research, these two components appear to be independent of each other. RAN is a skill composed of several factors, such as attention, perception, concept formation, memory, phonology, semantics, and motor skills. Difficulties with RAN are related to reading difficulties in several ways. One explanation is that deviations in the magnocellular system will lead to slower processing of visual stimuli, which in turn will lead to slower identification of letters. Another explanation is that RAN deficits can be an indication of a general dysfunction when it comes to processing in visual, auditive, and motor domains, in addition to the orthographic and phonological processing systems. When difficulties with PA and RAN are both present, the dyslectic impairments seem more pervasive and severe [21].

These works reflect the fact that the biological approach to dyslexia was re-addressed in international research.

#### 1.3.3. Neuroanatomical Aspects

##### Deviant Hemispheric Dominance of Language

Again, dyslexia attributed to deviant laterality in terms of the hemispheric dominance of language was in focus. The theories whereby elevated testosterone content in the mother during pregnancy affects the pattern of brain asymmetry received much attention [22,23,24,25]. The theories were seen as controversial but became a source of inspiration for looking at dyslexia in new ways. In the 1990s, new techniques were adopted in brain research, such as electrophysiological measurements (EEG) and brain scans (MRI). This allowed one to “see” depictions of the brain structure and function under given conditions.

These theories were, to some extent, exploited educationally. The balance model of dyslexia describes the relationship between reading and the function of the two brain hemispheres [26]. Beginner reading is primarily controlled from the right hemisphere of the brain but shifts to the left half of the brain as the child learns to read. In some children, this shift does not occur, and they develop a type of dyslexia in which they continue to rely on a right-hemispheric visuo-perceptual analysis of writing (P-type dyslexia). In other children, the shift over to the left hemisphere occurs prematurely, and they develop a type of dyslexia in which the visual recognition of writing is not sufficiently automated (L-type dyslexia). These different types of dyslexia must have different training programs, which are called hemisphere-specific stimulation [27].

##### The Hypothesis of Deviations in the Cerebellum

If reading and writing are to become functional, the skills must be automatized. The cerebellum is involved in the coordination of motor skills, balance, and muscle tone, but it is also involved in the automation of motor skills and in adapting the control of learning. According to Fawcett and Nicolson, cerebellum damage has led to difficulties with attention and working memory and with dyslexic symptoms when reading [28].

##### The Theory of Deviant Temporal Processing

Temporal processing implies the time spent to perceive, interpret, and produce sequential visual or auditory stimuli. Language is produced in sequences, and reading and writing are also activities that require similar temporal processing.

According to Tallal’s theories of auditory processing, the ability to distinguish different sounds in a fast tempo is a crucial linguistic building block [29,30]. A brain that does not distinguish quickly enough does not have the ability to distinguish between language sounds, which is a prerequisite for a child to learn to read. Hence, deviations in one or more of these processing skills can cause reading to degrade [31,32].

Additionally, deviations in the transient visual system, which reveals movements and changes in figures, is considered important for transmitting what one sees peripherally when reading [33]. The hypothesis of deviations in the magnocellular system comes from observations made by people with dyslexia who say that letters seem to move around or lie over each other [34].

##### New Definitions

The notion of the relationship between language and dyslexia showed that phonological problems were a common feature in people with reading/writing difficulties, regardless of the IQ goals [35,36]. An interesting and important debate regarding dyslexia began in the mid-1990s with a definition of dyslexia proposed by The Orton Dyslexia Society Research Committee in 1994:

*“Dyslexia is one of several distinct learning disabilities. It is a specific language-based disorder of constitutional origin characterized by difficulties in single word decoding, usually reflecting insufficient phonological processing. These difficulties in single word decoding are often unexpected in relation to age and other cognitive and academic abilities; they are not the result of generalized developmental disability or sensory impairment. Dyslexia is manifest by variable difficulty with different forms of language, often including, in addition to problems with reading, a conspicuous problem with acquiring proficiency in writing and spelling”*

[37]

The discrepancy criterion was no longer found valid, which was particularly clarifying when it came to the group with measured IQ between 70 and 85—the so-called “garden-variety” group. Low IQ could no longer be used as an exclusion criterion from being diagnosed with dyslexia. In his article “Towards a Definition of Dyslexia”, Lyon (1995) describes this as a theory-driven working definition that should be changed in accordance with research.

The fact that the field was constantly changing is shown by a definition that emerged from the British Dyslexia Association (BDA) three years later:

*“Dyslexia is a complex neurological condition which is constitutional in origin. The symptoms may affect many areas of learning and function, and may be described as a specific difficulty in reading, spelling and written language. One or more of these areas may be affected. Numeracy, notational skills (music), motor functional and organisational skills may also be involved. However, it is particularly related to mastering written language, although oral language may be affected to some degree”*

[38]

In line with both the WFN and the Orton Society/Lyon definitions, BDA 1998 defines dyslexia as an innate difficulty. Reading, spelling, and writing are described as areas of difficulty, but in addition, there are difficulties that clinicians recognize, namely comorbid difficulties with mathematics, motor skills, attention, and with interpreting symbols, such as sheet music.

These definitions show that the view of what dyslexia is changed with increased research-based knowledge. In many ways, one can say that it was moved from exclusion to inclusion. In the BDA definition from 1998, comorbid difficulties could be integrated in the dyslexia picture, where they previously served as exclusion criteria. On the one hand, researchers may see these additions as disturbing to their research. On the other hand, these changes have brought research and clinics closer together.

##### A Causal Model

The many theories about what dyslexia is and how dyslexia can be explained have led to a lot of uncertainty and disagreement among researchers, clinicians, in the school system, and, not least, in those concerned. One conclusion of this divergence is that there are several theories that can explain dyslexia, but no one theory alone can give an unambiguous explanation.

**Figure 1:**
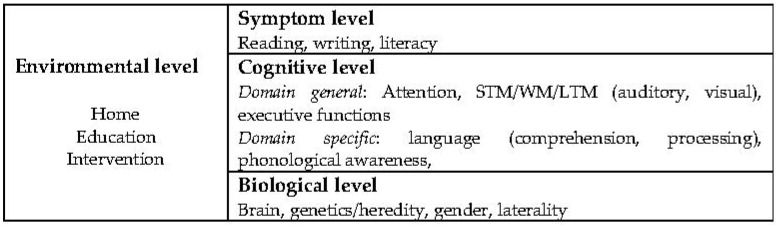
Basic causal model. From Morton and Frith [39]. Notes: levels (in bold) refer to the original model form Morton and Frith [39]. Descriptions of each level from Helland [40].

The causal model put forth by Morton and Frith provided a structural approach to several types of deviations [39]. Figure 1 illustrates their four basic interactive levels of explanation. The symptomatic level concerns observable behavior, to be understood at the cognitive level by underpinnings that have to be tested out, and which, again, can be understood or based on known or anticipated biological factors. Further, the environmental level implies the culture in which the person grows.

Frith elaborated on this model by focusing on the different aspects of dyslexia as a result of a phonological failure, of a magnocellular abnormality, of a cerebellar abnormality, and dyslexia with attention deficit disorder [41]. She also points out that more knowledge is needed when it comes to information-processing mechanisms, which can include difficulties with visual, auditory, or temporal processing. Accordingly, there should be no conflicts between the theories at the biological level, such as theories of failure of the magnocellular system, and theories at the cognitive level. What this model requires is valid and reliable assessment at all levels, as well as good clinical judgement. Not only the flexibility of the model, as demonstrated by Frith, but also its structural approach have made it sustainable and classical, not least in assessments of learning disabilities.

### 1.4. 2000–2020, Our Millennium

#### 1.4.1. 2000–2010, New Definitions

A cross-cultural study led by the Italian scientist Paulesu and his international team introduced exciting and challenging dyslexia research by the new millennium [42]. The study focused on whether dyslexia differs in different languages and cultures. An international group of students with dyslexia was tested at the four levels described by Morton and Frith 1995, and modern brain scanning methods were applied. It was concluded that the underlying cognitive and biological factors in people with dyslexia are universal.

Similar views were promoted by the English researcher Elaine Miles [43]. She pointed to the dominant role the Anglo–American language has played in dyslexia research and stressed the importance of bringing out research in different languages. This applies not only to the various orthographic features of the written languages, such as the degree of transparency or depth, but also to the role that linguistic inflection patterns, tonality, and logographic fonts can play in how we define dyslexia. This was also reflected in studies of variations in Chinese dyslexia [44]. Other researchers underlined that writing is as equally important a factor in dyslexia as reading and that cognitive skills that are known to be essential in reading also affect writing [45]. These new approaches were mirrored in concurrent definitions—first, in the definition by the International Dyslexia Association (IDA) in 2002:

*“Dyslexia is a specific learning disability that is neurobiological in origin. It is characterized by difficulties with accurate and/or fluent word recognition and by poor spelling and decoding abilities. These difficulties typically result from a deficit in the phonological component of language that is often unexpected in relation to other cognitive abilities and the provision of effective classroom instruction. Secondary consequences may include problems in reading comprehension and reduced reading experience that can impede growth of vocabulary and background knowledge.”*

[46]

Five years later, a new BDA definition used the terms “literacy” and “language related skills” and included not only cognitive factors but also intervention, with a special focus on digitalization and the role of the teacher:

*“Dyslexia is a specific learning difficulty which mainly affects the development of literacy and language related skills. It is likely to be present at birth and to be lifelong in its effects. It is characterised by difficulties with phonological processing, rapid naming, working memory, processing speed, and the automatic development of skills that may not match up to an individual’s other cognitive abilities. It tends to be resistant to conventional teaching methods, but its effects can be mitigated by appropriately specific intervention, including the application of information technology and supportive counselling”*

[47]

That comorbidity had been ignored or avoided in research by studying “pure” groups was criticized. Due to the relationship between speech, language, and reading disorders, it was argued that to understand these disorders fully, the relationships between them must be considered both on a cognitive and etiological basis [48]. A few years later, the working definition presented in the Rose Report identified the cognitive factors typical of dyslexia and identified dyslexia as a continuum rather than a binary impairment [49]:

*“Dyslexia is a learning difficulty that primarily affects the skills involved in accurate and fluent word reading and spelling. Characteristic features of dyslexia are difficulties in phonological awareness, verbal memory and verbal processing speed. Dyslexia occurs across a range of intellectual abilities. It is best thought of as a continuum, not a distinct category, and there are no clear cut-off points. Co-occurring difficulties may be seen in aspects of language, motor coordination, mental calculation, concentration, and personal organization, but these are not, by themselves, markers of dyslexia. A good indication of the severity and persistence of dyslexic difficulties can be gained by examining how the individual responds or has responded to well-founded intervention”*

[49]

#### 1.4.2. 2010–2020

In their summary of the current understanding of dyslexia, Peterson and Pennington concluded that much progress had been made in understanding dyslexia [50]. It is a brain-based and neurodevelopmental disorder, multifactorial and universal in nature. Advanced use of brain scanning methods and analyses, as suggested in EEG, and of longitudinal design using fMRI have enhanced the understanding of dyslexia [51,52,53]. Additionally, modern eye tracking methods have shed further light on how the dyslexic brain works during reading [54]. Along with these research methods, frequent comorbidities with other learning disabilities, such as ADHD, language, and mathematic impairment, are in focus [55]. Additionally, the dominance of the Anglo–American language, and hence, problems related to deep orthography, which may have biased the understanding of dyslexia, was questioned in multicultural studies [56,57,58,59,60].

Thus, currently, dyslexia is seen as a complex disorder that varies from person to person, building on the biological, linguistic, psychological, and pedagogical knowledge. According to Snowling, Hulme, and Nation, we have still not reached a consensus on what dyslexia is. Rather, they argue that:“ …loosening the criteria for dyslexia has influenced common understanding of the condition and led to diagnostic confusion [61]. In the longer term, the use of the term may need to change …… that loosening the criteria for dyslexia means that a far wider range of individuals now receive the label; furthermore, by understanding the cooccurrence of dyslexia with other disorders, we reach a better understanding of the heterogeneity of its manifestations” (p. 501). Further, they conclude that this multi-dimensionality of dyslexia is complex, but to fail to acknowledge this complexity cannot be defended ethically.

With this connotation of dyslexia, a definition recently codified in U.S. law (PL 115–391) is not promising. Here, dyslexia is defined as

*“an unexpected difficulty in reading for an individual who has the intelligence to be a much better reader”*, as referred to in Shaywitz [62].

This is definitely loosening the grip of any evidence-based understanding of dyslexia, as it is pointing back to the WNF definition of 1968.

### 1.5. Evaluation of the Different Definitions

Applying the model by Morton and Frith [39], only two of the definitions reported here meet its requirements. The WNF definition (1968) denotes a symptom (difficulty in learning to read), cognitive traits (adequate intelligence), biological traits (constitutional origin), and environment (sociocultural opportunity). However, as dyslexia is seen as a multifactorial disorder, the four levels are inadequately described in this definition. The second definition that reaches the requirements is that of the BDA [47], which defines symptoms (affects the development of language and literacy skills), the cognitive level (difficulties with phonological processing, rapid naming, working memory, processing speed, and the automatic development of skills that may not match up to an individual’s other cognitive abilities), the biological level (present at birth and to be lifelong in its effects), and environmental level (effects can be mitigated by appropriately specific intervention, including the application of information technology and supportive counselling). Four of the definitions include comorbidities [37,38,47,49], while two include intervention [47,49]. The latest definition codified in U.S. law (PL 115–391) only includes reading and intelligence at the cognitive level.

## 2. Part II—Dyslexia Publications 1950–2020

To further assess the development of dyslexia research, a database search of scientific publications on dyslexia during the period 1950 to 2020 organized by decennium (1950–1960, 1960–1970, 1970–1980, 1990–2000, 2002–2010, 2010–2020) was executed.

Due to its inclusiveness, Google Scholar was preferred as a search engine over more selective databases (PubMed, Eric, Scopus, APA PsycNet). The results of the count searches were generated in three steps: first, basic counts on the terms “dyslexia” and “dyslexia definition”; second, counts by levels (symptomatic, cognitive, biological, and environmental) and areas (sensorimotor, comorbidities); third, counts within the levels and areas using central terms from dyslexia research, and especially from the BDA 2007 definition. The searches indicate the frequencies of attention in the publications and not purely the inclusiveness of the selected search term. This means that, in the counts, there may be multiple hits or citations within the same search. However, the goal of the searches was to assess what has been focused on during the period 1950–2020, irrespective of the publication quality (peer review) or journal index scores. Consequently, the counts cannot be statistically analyzed; they are instead seen as indications of how dyslexia focus has developed.

All categories of search words and related pop-up search words presented by Google Scholar are shown in Table 1.

The left panel contains the three main categories of the search: A. Main categories (dyslexia, dyslexia definitions); B. Level (symptomatic, cognitive, biological, environment); C. Areas (sensorimotor, comorbidities). Each of the B and C categories is then itemized into frequently used subtypes (middle panel). To ensure inclusion of the earliest research, the searches were based on terms used in the first decennium, 1950–1960. Items at B Level: symptomatic (3: reading, writing, literacy); cognitive (3: auditory, visual, phonological); biological (4: brain, gender, genetics, laterality); environment (3: home, education, intervention). Items in C area: sensorimotor (3: motor, hand, eye); comorbidities (4: LI/DLD, mathematics, Dyslexia in L2, ADHD). The right panel shows the algorithm pop-up lists for each item. To control for any effect of the algorithms, all searches were conducted in one day in April 2022. As the lists show, the categories are not mutually exclusive.

Table 2 shows the counts of the A. Main category searches on “dyslexia” and “dyslexia definition”. As expected, there is a substantial increase in publications. However, the interesting part is that relatively few of the publications include or focus on a definition of dyslexia, although an increase was seen in the decade 2010–2020.

As can be seen in Table 3, there was a substantial increase in publications from 1950 to 2020 across the four levels and the two areas. Additionally, a relative change in the focus of interest was seen when comparing the levels in particular. Most publications were at the symptomatic level across all decennia, here ranged as number 1. Range number 2 alternated between the cognitive level and the environmental level, where the increase in publications at the environmental level was remarkable during the period 2010–2020. Although publications at the biological level remained steady (ranged as number 4), there was a substantial increase in publications at this level from 2000 on.

Regarding the two areas, the 1950 to 1970 period started out with a dominance of sensorimotor publications but was bypassed by the publications on comorbidities from 1970 onward.

To assess the four levels and two areas further, the benchmark functions within each level and area were first counted by decennium. Next, the relative contribution measured in percent by decennium was calculated. Again, and in the thread with the earlier description of the counts, this does not add to accuracy but contributes to a depiction of the relative development or change within the levels. The hit words from each level are seen in Figure 1 but were adapted to the most frequently used terms from 1950 onward associated with each level and area. The searches were all combined with “dyslexia” as the first word and selected items within each of the levels and areas as the second word, as shown in Table 2, middle panel. As can be seen, the starting point in 1950 was a few publications within all the items, with a steady growth by 2020.

**Figure 2:**
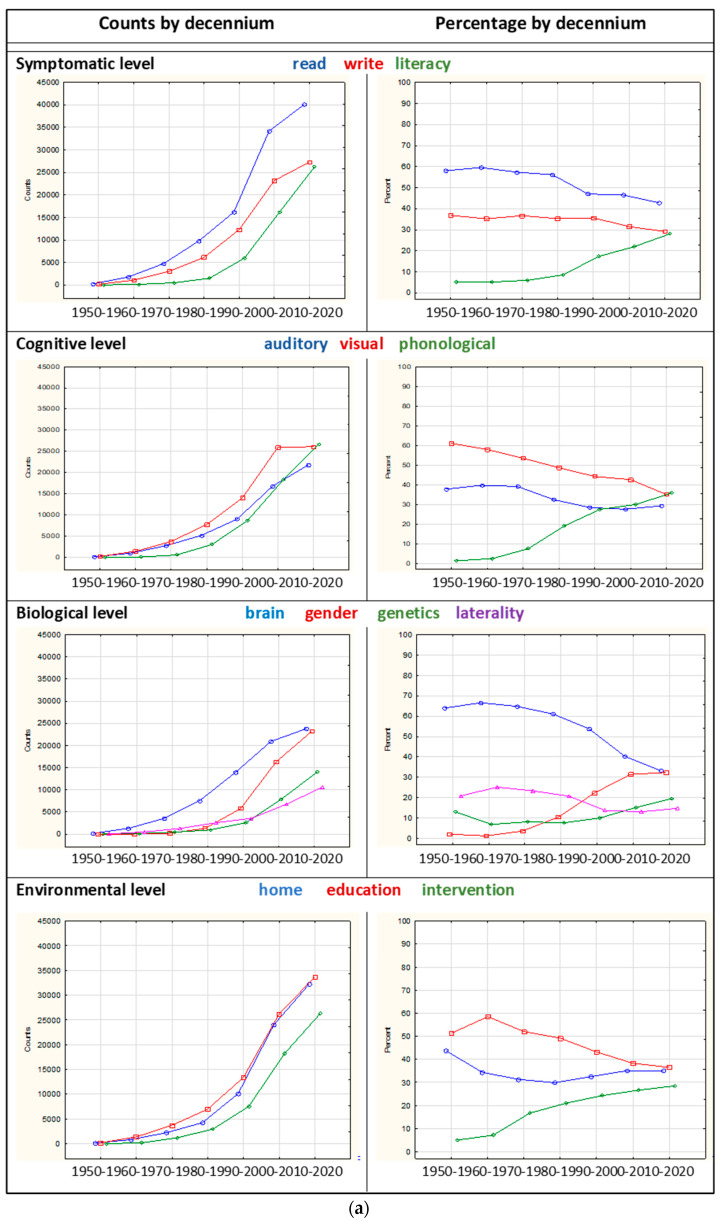

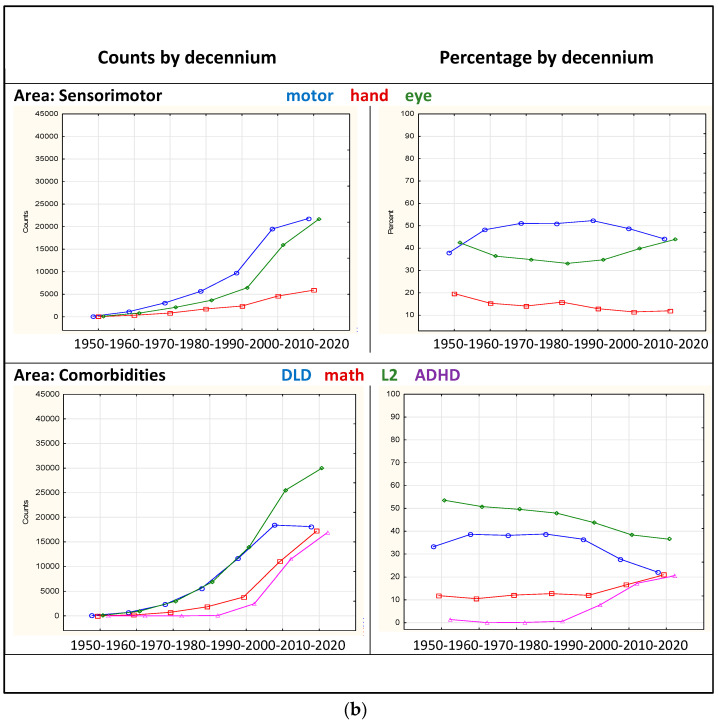
(**a**) Counts and percentage by decennium at the four levels and two areas. (**b**) Counts and percentage by decennium at the two areas.

The right panels of Figure 2a,b illustrate the relative change by decennium. At all four levels (Figure 2a), there is a change in focus around 1980. At the symptomatic level, “literacy” has been increasingly used. At the cognitive level, the terms “Visual” and “auditory” dyslexia were used. However, from 1970 onward, a substantial increase in “phonological” is seen, with relatively as large a focus as the other two terms. At the biological level, relatively, most publications were on the “brain”, while “gender” increased to be at the relatively same level as the “brain”, while the relative contribution of “genetics” and “laterality” show little increase. Finally, at the environmental level, the focus on “home” and “education” has been relatively steady, while the focus on “intervention” increased from around 1980 onward. In sum, this tentative comparison showed a shift in the focus toward more interest in “literacy” over foremost “reading” but also “spelling” at the symptomatic level,, on “phonology” at the cognitive level, on “gender” at the biological level, and on “intervention”.at the environmental level. 

Additionally, as can be seen in Figure 2b, left panel, the two “areas” of sensorimotor and comorbidities have seen an increase in publications. The right panel shows the relative changes in focus. Relatively, the sensorimotor area publications on handedness have remained low, while the publications on eye have stayed in the middle position, and the publications on hand showed a decrease and then an increase. Within the comorbidities area, the publications on language impairment increased gradually from 1990, and on second language learning and AHD—from 2000.

## 3. Discussion

Along with the general increase in research, publications on dyslexia have increased accordingly during the period 1950 to 2020. Although reading research has played the main role during the whole period, thematic shifts have been seen over these 70 years. In particular, a substantial shift was seen by the turn of the millennium from an Anglo–American leadership, both on the research and understanding, to a broader cross-cultural and cross-lingual approach. The insights into the cognitive underpinnings have developed from sorting into groups to a complex dimensionality. Advanced use of imaging technology has revealed brain functions and structures typical of dyslexia, and digitalized methods of intervention are about to replace the role of the teacher.

In short, dyslexia research has grown, and the understanding of this impairment and how to deal with it has changed both from a historical point of view and from what the publication counts reveal. According to Peterson and Pennington, it is supposed to be one of the best understood and most extensively studied learning disorders [50]. Interestingly, the historical overview illustrates how attention on dyslexia benchmarks moves in spirals: new knowledge discharges old knowledge then to be restored and reused. Terms such as “visual”, “auditive”, and “phonological” and “laterality”, have been redefined across cultures and recirculated by new methods of assessment (see, e.g., Refs [63,64,65,66,67,68]).

Since the first medical publications on “word blindness”, many different professional groups have engaged in dyslexia research. As the pop-up items in Table 1 show, it is a complex and multifactorial deficit across levels, which makes it difficult to find an unambiguous causal relationship. Consequently, as shown in the first part of this paper, there is still no agreement on how to define dyslexia.

This may explain why few publications on dyslexia identify or base their work on a particular *definition*. This may also be the reason why central researchers have discussed the idea of loosening up the criteria of dyslexia or using a very problematic, vague definition, as proposed in the U.S. [61,62]. This lack of definitional criteria as a basis for participant and method selection should attract much more focus in future studies. The causal model by Morton and Frith has proven to be robust, coherent, and a useful approach to many different psychological conditions [39]. Thus, in line with Frith’s examples of the adaptability of the model, its framework was used to evaluate the different definitions presented earlier in this paper [41]. By this procedure, the BDA 2007 stood out as a valid definition to be used as a basis for the multifactorial impairment of dyslexia. The structure, and yet, the flexibility of the model, open up new findings and theories, which, in turn, can appraise future proposals for definitions.

According to the model, information is needed at all four levels. The symptomatic level is what we can observe: literacy at a pre-, emergent, and functional stages. The cognitive level encompasses more than the three terms “auditory”, “visual”, and “phonological” used in Table 2. At the biological level, dyslexia research relates to innate factors, where some can easily be observed and assessed, while other factors need advanced testing procedures. The interaction between these three levels and the fourth environmental level is detrimental to literacy outcome in dyslexia. According to Frith, impairment at the symptomatic level cannot alone identify dyslexia [41]. The cognitive level is called the “bridge” between the symptomatic and the biological level. Impairments within two or more dyslexia benchmarks at the cognitive level combined with literacy problems point to “true” dyslexia. In contrast, deficits at the symptomatic level only denote “false” dyslexia.

The BDA 2007 definition includes digital support as a valid method of intervention. This was perhaps of greater importance in 2007 than currently. The accelerating multitasking, the universal use of smartphones, social media, and digital aids may mask the need for individually adjusted teaching essential to dyslexia. It is assumed that digital aids, such as spell checks and voice control, are of great help for many persons with language difficulties and dyslexia. Typically, impairments within the working memory, processing speed, and attention are not necessarily compatible with the digital diversity [69].

This brings up a flashback to the sensorimotor area and the theories and practices of Orton [4]. Handwriting is called the “language by hand” and is important for the early recruitment in letter processing in the brain. According to James and Engelhardt, handwriting may facilitate reading acquisition in young children [70]. A recent large metastudy concluded that there is an association between non-right handedness and reading and language impairments due to “shared biological pathways underlying brain lateralization, handedness, and cognitive functions” [63]. This points to research on keyboard writing using both hands and assessing if this has any long-term effect on language lateralization of the brain.

As we have seen, comorbidity has been used both as exclusion and inclusion criteria in the different definitions. At the cognitive level, the classical multiple-component model of working memory illustrates how language and literacy skills are dependent on the two loops, the phonological and the visuo-spatial, with consequences to the functions of the central executive [71,72]. As in ADHD, impairments within these functions are seen in dyslexia [73,74,75]. LI/DLD affects many aspects of language learning, not least L2 acquisition. Regarding mathematics, difficulties can, among other things, be of a linguistic nature concerning the concepts, terms, transcoding of written problems, according to DSM-IV [76].

The counts shown in Table 3 indicate that research within the sensorimotor area has gained relatively less attention compared to the comorbidity area, but with the digital shift, this may change, as mentioned above.

As illustrated in the left panels of Figure 2, there has been a substantial increase in publications at all four levels and in the two areas, as counted by Google Scholar. The relative change is illustrated in the right panel, showing that the increase in publications has leveled out earlier due to thematic disproportions. The change took place mainly around 1980–1990, manifesting that the “pioneers” were trailblazers for later research. Interestingly, the sensorimotor area does not show the same pattern, as the counts of “hand” did not increase. However, as we have seen, there is a renewal in interest in the field of lateralization.

## 4. Summary

The introduction to this paper gave a brief review of some curious cases of dyslexia detected in gifted boys, described by doctors in medical journals. Today, dyslexia is a commonly known term and denotes an innate disposition of impaired reading, writing, and hence, literacy, in a digitalized society with rapidly increasing and changing demands for literacy. We know of top politicians, businesspeople, criminals, artists, academics, or the unemployed who talk openly about being dyslexic, a development brought about through much clinical work and research evolving from the 1950s.

Literacy skills develop with age and learning. Frith separated this development into three stages: the preliteracy stage, before formalized literacy training has started; the emergent literacy, when the child receives formal literacy training; the literacy stage, when reading and writing have become tools for academic achievements [41]. In the first publications on dyslexia research, “reading” and “writing” (or “spelling”) were focused on. The increased use of the more holistic “literacy” term denotes that dyslexia entails more than decoding problems but has a developmental aspect, where each stage has its own characteristics.

Nevertheless, the “winner” of all in terms of the counts is the term “read” within the symptomatic level. Until the turn of the millennium, most research was within orthographies using the Latin alphabet, mainly dominated by the two deep Anglo–American orthographies. The findings did not always harmonize with what was found in other Latin orthographies [58,77,78,79], which also led to much reading research in other orthographies [80,81].

Comparatively, there are fewer studies on *writing*. Often, writing is not accounted for in dyslexia studies, which is surprising, especially to teachers. However, the interpretation of a written text is less accurate, as it involves more than coding words. From a researcher’s point of view, factors such as, e.g., motor control, vocabulary, intellectual abilities, interests, motivation involved in writing, may clutter both the testing and interpretations. In this respect, studies using keystroke logging during text writing are promising [82,83,84].

From the 1950s to the 1970s, dyslexia was either not known or not acknowledged in schools, and students who did not learn to read or write were often labeled with the word “retarded”, which is now seen as inappropriate. Some were sent to special schools or placed in special classes. The idea of integration and adjusted teaching has improved the situation of students with dyslexia greatly. However, there are still reports of relatively many prison inmates with dyslexia and with little schooling [85].

Well-designed longitudinal studies on developmental research and RCT studies on the effects of intervention have long been considered the gold standard but are, for many reasons, demanding and often difficult to carry through, consequently rounding off with conclusions needing several reservations. Some studies have successfully followed at-risk groups of children from early childhood onward, finding usable methods for prevention and intervention [51,75,86]. Such studies are important to the present and future understanding of and intervention in dyslexia. Dyslexia is now commonly addressed as an endophenotype impairment with individual variations rather than identified at a group level. This, however, is demanding, requiring standards of evidence-based practice based on the current knowledge on education and cognitive neuroscience [87]. In this respect, the four-level model by Morton and Frith has proven to be a valid basis as an outline for dyslexia definitions [39]. The BDA 2007 definition does not only reach this standard, but it also includes comorbidities, which earlier on were used as exclusion criteria. It is a challenge for future research to continuously use and possibly redefine dyslexia definitions in line with such standards.

## Figures and Tables

**Table 1:** Search words. Main categories, levels and areas.

A. Main Categories	Pop-Up Items	
**Dyslexia**	*Developmental, phonological, reading, dysgraphia, dyscalculia, spelling, comorbidity, dyslexia friendly, multisensory, assistive technology, dyspraxia, auditory processing, phonological awareness, dyslexia friendly schools*	
**Definitions**	*Developmental dyslexia, phonological dyslexia, dyslexia reading, dyslexia dysgraphia, dyslexia dyscalculia, dyslexia spelling, dyslexia comorbidity, dyslexia friendly*	
**B. Level**	**Pop-Up Items**	
**Symptomatic**	*Developmental, symptoms, phonological, reading difficulties, children with reading, learning difficulties, occupation choices*	
	reading	comprehension, strategies, difficulties, disability, problems, interventions, fluency, ability, programs
	writing	problems, difficulties, strategies, instruction, skills, interventions, system, spelling, disability
	literacy	psychological assessment, and inclusion, difficulties, interventions, problems, development, strategies, skills, learning
**Cognitive**	*Developmental cognition, cognitive subtypes, cognitive analysis*	
	auditory	memory, brain, processing, processing disorder, discrimination, working memory, attention, cortex
	visual	processing difficulties, perception, developmental, spatial, deficit, word, auditory, visual word form area
	phonological	awareness, deficit, processing, theory, deficit hypothesis, loop, intervention, skills, training, memory
**Biological**	*Developmental, causes, biological basis, psychiatry, biological unity*	
	brain	function, imaging, developmental, fMRI, acquired, phonological processing, regions, scans
	gender	differences, ratio, age, brain imaging, specific, prevalence, bias
	genetics	review, chromosomes, MRI
	laterality	brain function, imaging, developmental, fMRI, acquired, phonological processing, regions, scans
**Environmental**	*Developmental, home literacy, classroom, least restrictive, virtual, genes, assistive technology, inclusive, environment matters*	
	home	literacy environment, homework, homelessness, home schooling, homework strategies
	education	equality act, higher education, employment, in adults, in context higher education, students
	intervention	programs, strategies, in the classroom, for children, review, phonological awareness, meta-analysis, studies, program
**C. Area**		
**Sensorimotor**	*Impairments, synchronization, integration*	
	motor	skills, coordination, deficits, impairment, cerebellum, familial, development, difficulties, adults
	hand	Handedness, meta-analysis, developmental dyslexia, left handedness
	eye	motor skills, motor coordination, motor deficits, motor impairment, cerebellum motor, familial dyslexia, motor development, children with dyslexia motor function, motor difficulties adults with dyslexia
**Comorbidities**	*Adhd, autism, dyscalculia, defining comorbidity, dyspraxia, dyslexia, specific language impairment, dysgraphia*	
	LI/DLD	developmental, auditory processing, family risk, specific reading disability, adolescence, preschool, literacy outcomes, longitudinal investigation
	mathematics	math strengths, math abilities, developmental
	dyslexia in L2	acquisition, learning, reading, intervention, teachers
	ADHD	executive, dyscalculia, dyspraxia, gifted, cerebellum, phonological dyslexia

LI: Language impairment; DLD: Developmental Language Disorder.

**Table 2:** Counts, publications on search words: “Dyslexia” and “Dyslexia Definitions”.

Decennium	Dyslexia	Definition
2010–2020	57,300	22,500
2000–2010	57,900	17,700
1990–2000	21,400	9230
1980–1990	13,200	4420
1970–1980	6490	2100
1960–1970	2570	615
1950–1960	471	69
**Total**	**159,331**	**56,634**

**Table 3:** Counts: Levels and Areas.

Decennium	Levels								Areas			
	Sympt		Cogn		Biol		Env		SM		Comorb	
	Counts/Range		Counts/Range		Counts/Range		Counts/Range		Counts/Range		Counts/Range	
2010–2020	93,700	1	74,600	3	71,600	4	92,300	2	49,420	2	82,200	1
2000–2010	73,600	1	61,100	3	51,880	4	68,400	2	40,000	2	66,400	1
1990–2000	34,480	1	31,690	2	25,810	4	31,030	3	18,560	2	31,790	1
1980–1990	17,410	1	15,920	2	12,340	4	14,170	3	11,060	2	14,448	1
1970–1980	8284	1	6961	3	5478	4	7150	2	6055	2	6074	1
1960–1970	3037	1	2384	2	1967	4	2359	3	2343	1	1849	2
1950–1960	448	1	313	2	283	4	302	3	356	1	211	2
**Total**	**230,959**	**1**	**192,968**	**4**	**169,358**	**3**	**215,611**	**2**	**127,794**	**2**	**202,972**	**1**

## Data Availability

Not applicable.
